# A LightGBM-Based EEG Analysis Method for Driver Mental States Classification

**DOI:** 10.1155/2019/3761203

**Published:** 2019-09-09

**Authors:** Hong Zeng, Chen Yang, Hua Zhang, Zhenhua Wu, Jiaming Zhang, Guojun Dai, Fabio Babiloni, Wanzeng Kong

**Affiliations:** ^1^School of Computer Science and Technology, Hangzhou Dianzi University, Hangzhou, Zhejiang 310018, China; ^2^Industrial NeuroScience Lab, University of Rome “La Sapienza”, Rome 00161, Italy

## Abstract

Fatigue driving can easily lead to road traffic accidents and bring great harm to individuals and families. Recently, electroencephalography- (EEG-) based physiological and brain activities for fatigue detection have been increasingly investigated. However, how to find an effective method or model to timely and efficiently detect the mental states of drivers still remains a challenge. In this paper, we combine common spatial pattern (CSP) and propose a light-weighted classifier, LightFD, which is based on gradient boosting framework for EEG mental states identification. The comparable results with traditional classifiers, such as support vector machine (SVM), convolutional neural network (CNN), gated recurrent unit (GRU), and large margin nearest neighbor (LMNN), show that the proposed model could achieve better classification performance, as well as the decision efficiency. Furthermore, we also test and validate that LightFD has better transfer learning performance in EEG classification of driver mental states. In summary, our proposed LightFD classifier has better performance in real-time EEG mental state prediction, and it is expected to have broad application prospects in practical brain-computer interaction (BCI).

## 1. Introduction

Fatigue driving is an important cause of traffic accidents. According to data from U. S. National Transportation Safety Board, the annual economic losses caused by driving accidents in the United States are more than $12.5 billion [1]. Fatigue has no obvious symptoms but usually manifests as lethargy, fatigue, or weakness [2]. Therefore, developing technologies to monitor and predict driver' mental state or the ability to safely drive the vehicle will have significant social and economic benefits [3].

At present, for fatigue driving detection, the academic community has carried out a lot of research work. To sum up, it mainly lies in the following aspects: (1) mental activity testing using response time and accuracy by passive BCIs [4, 5], which mainly perform an assessment of a subject's cognitive states [6, 7], (2) detection of eye movement parameters, such as eye squint movement, percentage closure of eyes (PERCLOS) [8], and so on, (3) active detection by means of questionnaires, (4) sensor-based methods to find some fatigue indicators by steering force (steering grip pressure), skin conductance, blood volume pulse (BVP), and so on [9, 10], and (5) performing fatigue state detection by bioelectrical signals, such as EEG, EOG (electrooculogram), EMG (electromyogram), and ECG (electrocardiogram) [11–16].

For physiological-electric-based detection, researches have shown that these signals have a strong correlation with the driver's mental state, so these signals can be more accurate to detect driving fatigue. Among the above various researches of fatigue detection, EEG analysis methods are considered to be most convenient and effective for its good time resolution and sufficient spatial resolution. It is known that EEG represents the brain activity by the electrical voltage fluctuations along the scalp [17]. As an effective tool for the indirect measurement of neural activity, EEG is widely used in neuroscience, cognitive science, cognitive psychology, and psychophysiology research, etc. On the other hand, driving behavior involves a variety of behaviors, such as motions, reasoning, audiovisual processing, decision making, perception, and recognition, which is also affected by emotions, attention [18], and many other psychological factors. These physical and mental activities related to driving are reflected in EEG signals.

In recent years, a number of methods for fatigue detection using EEG have been proposed; for example, Kar et al. [2] investigated a number of fatigue-indicating parameters based on higher-order entropy measures of EEG signals in the wavelet domain. In particular, they present a method based on a kind of entropy measures on the EEG signals of the subjects for the relative quantification of fatigue during driving. Charbonnier et al. [19] proposed an online innovative EEG index and proved that the proposed index can be used based on the alpha activity to effectively assess the operator's mental fatigue status. Roy et al. [20] applied a Fisher's linear discriminant analysis (FLDA) to detect and classify EEG-based mental fatigue. In [21], the authors used a KPCA-SVM classifier to distinguish between normal and fatigue mental state, with an accuracy rate of 98.7%. Maglione et al. [22] used high-resolution EEG and neurophysiological variables to analyze the increase in cerebral workload and the insurgence of drowsiness during car driving and acquired a workload index. In 2014, Zhang et al. [23]presented a real-time method with various entropy and complexity measures for the detection and identification of driving fatigue from EEG, EMG, and EOG signals, and the accuracy of estimation is about 96.5%–99.5%. Appriou et al. [24] presented a comparison of 4 modern machine learning algorithms in order to compare EEG-based workload level classification performances and found that CNN can obtain better performance (mean = 72.7% ± 9.1) than a LDA classifier with CSP spatial filters in classifying two workload levels (low vs. high) for both user-specific and user-independent studies. In [25], the authors developed an adaptive stacked denoising auto encoder (SDAE) to tackle cross-session mental workload (MW) classification task, and the adaptive SDAE is also demonstrated to be acceptable for online implementation. All together, these articles support the knowledge that mental fatigue can be efficiently detected by EEG with classification performances varying between 75% and 98%.

Other feature extraction and analysis methods are also used in mental state detection, such as EEG and fNIRS joint analysis [26], discrete wavelet transform [27], wavelet-packets transform (WPT) [28], integrating feature selection, and fusion on high-level EEG features from different models [29]. In recent years, deep learning-based models have also been used in mental state classification, for instance, deep convolutional neural networks [29], long short-term memory network (LSTM) [30], and switching deep belief networks with adaptive weights (SDBN) [31].

Although these methods have achieved excellent performance, how to design appropriate models to obtain robust, real-time, and high-accuracy classification performance of driving mental states by EEG still remains a challenge for a series of reasons. First, EEG shows the characteristics of instability and randomness, EEG signals collected by the single subject (intrasubject) or between two different subjects (intersubject) tend to have large differences over time [32]. Second, the low signal-to-noise (SNR) ratio of EEG often affects the accuracy of detection. Third, with the continuous improvement in EEG acquisition equipment, EEG signals gradually show multidimensional and complex features with a large time and space consumption during processing.

LightGBM [33] is a gradient boosting framework that uses a decision tree-based learning algorithms. It is distributed, efficient with faster training efficiency, and can handle a large amount of applications, but there also exists deficiencies when dealing with high-dimensional features for EEG signals, like lower accuracy, as well as time consumption. Therefore, in this article, we improve and design a LightGBM-based model, LightFD, which adopts the histogram-based decision tree algorithm and the leafwise leaf growth strategy with depth limitation to solve the problem of excessive xgboost memory consumption, which is more suitable for practical EEG clinical applications. Now, LightGBM has been applied to EEG signal classification and has achieved certain results in practical problems, such as emotion recognition [34, 35], epilepsy prediction [36], and so on.

Transfer learning methods have been widely used for EEG signal classification in recent years [37–40], which could transfer the previous extracting features in one kind of trained samples to another sample for some specific decision tasks. Due to its great advantages of lower time consumption, transfer learning can bring more practical application possibilities for EEG analysis.

Motivated by the advantages of LightGBM and following our previous work [41], where only a CNN-based model was investigated to realize the EEG-based binary classification of mental states, and the model is time consuming, moreover, the transfer learning capability of the model is not analyzed. Thus, in this article, we aim to design a LightGBM-based classifier, LightFD, to implement the light-weighted analysis of triclassification identification of EEG mental states, and furthermore, we will also test and validate the efficiency and robustness of LightFD in the aspect of transfer learning and compare them with those of manifold embedded distribution alignment (MEDA) [42] and metric transfer learning (MTLF) [43].

## 2. Materials

### 2.1. Subjects

We recruited 10 healthy subjects for EEG data collection. All of them were within 23 and 25 years old and possess Chinese manual driver C1 license. They were informed in advance of the entire experimental process and instructions and also required to keep calm without drinking irritating beverage, like coffee, alcohol, and so on before the experiment. All participants provided their written consents, and the research was approved by the ethics committee of our university.

### 2.2. Experimental Setup

**Figure 1:**
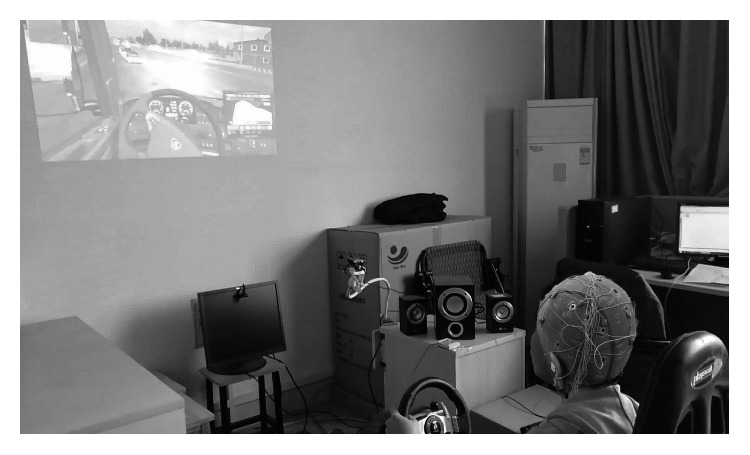
Driving simulation experiment platform.

To collect EEG data during driving, we constructed a simulation platform, as shown in Figure 1, which consisted of a racing seat cushion, steering wheel, liquid crystal display (LCD), speaker, video camera, and projector. A 16-channel gUSBamp amplifier (g.Tec Medical Engineering GmbH) was used to record EEG signal. Besides, two more computers were employed for (1) simulating the track with the special “Speed-Shift 2 Unleashed (NFS-S2U)” software, recording all the parameters during driving with “WorldRecord” software, and (2) collecting the video and sound stimuli, dealing with EEG signals, respectively [12, 41].

### 2.3. Experimental Protocol

The whole experiments lasted for two days and were conducted between 18 : 00 and 21 : 00 in a quiet and isolated environment. The first day was considered the practice stage for familiarizing with the track and stimulating software and experimental operations, and the second day was the formal experimental stage for collecting EEG data. The heart rate and blink were simultaneously collected with EEG by corresponding sensors, such as ECG electrode attached on the subject's wrist and video camera placed in front of the subjects, which were used to aid judgment in the level of mental states. According to previous studies [44, 45], the numbers of blink and heart rate in the situation of awake would be higher than those in the situation of drowsiness. Moreover, we counted the average number of blink and heart rate of all the subjects throughout the whole experiments. We found that at the beginning stages, when the subjects were only asked to drive a car at a predefined speed without any video or sound stimuli, the average number of blinks is above 20 times/min, up to 24 times/min, and the average heart rate was close to 90 times/min. As the stimuli were introduced into the experiments, the average number of blinks changed to 12–20 times/min, and the heart rate was 78–85 time/min. At the last stage, the subjects were not given any stimuli, the average number of blinks increased to 22 times/min, and the average heart rate was 73 times/min. Therefore, we divided the mental states into 8 stages: WUP, PERFO, TAV3, TAV1, TAV5, TAV2, TAV4, and DROWS [12, 41]; the detailed introduction of these eight stages is shown in Table 1.

**Figure 2:**
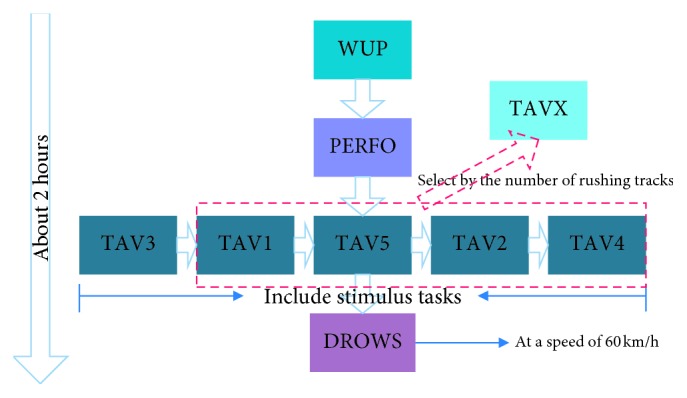
The schematic diagram of experiment procedure.

The flowchart of the experiments is shown in Figure 2. There were two kinds of driving tasks during the experiments: one was a simple driving task, which only required the subject to drive like the practice stage and did not exert any sound and video stimuli. This kind of driving tasks included three stages: WUP, PERFO, and DROWS. WUP was the beginning of the experiment with a baseline driving speed, and PERFO was similar to WUP but required the subjects to drive at a speed of 2% faster than WUP. DROWS was the last stage of the experiments with a fixed driving speed of 60 km/h. The other stages introduced additional video (“alert”) and sound stimuli (“vigilance”) to simulate situations such as red lights and traffic jams that might occur in real driving, which include five TAV stages: TAV1–5. All TAV stages were exerted with different stimulus frequencies of sound (“vigilance”) and video (“alert”) stimuli that appeared on the LCD screen 1 m ahead of the subjects, and the corresponding buttons were pressed by the subjects: LEFT button for “vigilance,” and RIGHT button for “alert.” Five TAV stages: TAV3, TAV5, TAV1, TAV2, and TAV4 were executed in sequence.

Because TAV3 is the first stage with video and sound stimuli, the subjects were bound to drive very carefully and complete the corresponding operations as quickly and accurately as possible, so they were in the most awake state. DROWS was the last stage without any stimuli. It just required the subjects drive with a fixed speed of 60 km/h. It seemed monotonous and boring, especially after about 2 h of driving; therefore, the subjects were extremely prone to fatigue in this stage. In addition, the obvious differences in blink and heart rate between TAV3 and DROWS further confirmed the correctness of the design of this experiment. Moreover, we defined a “neutral” stage as TAVX, which was neither fatigue nor awake. However, the time required for each subject to enter the fatigue state may be different, and TAVX is one of those 4 stages: TAV1, TAV2, TAV4, and TAV5, at which the number of rushing out of the track is closest to the average of rushing out of the track during the experiment. Accordingly, the collected data at TAVX were then used for analysis.

### 2.4. EEG Recording

**Figure 3:**
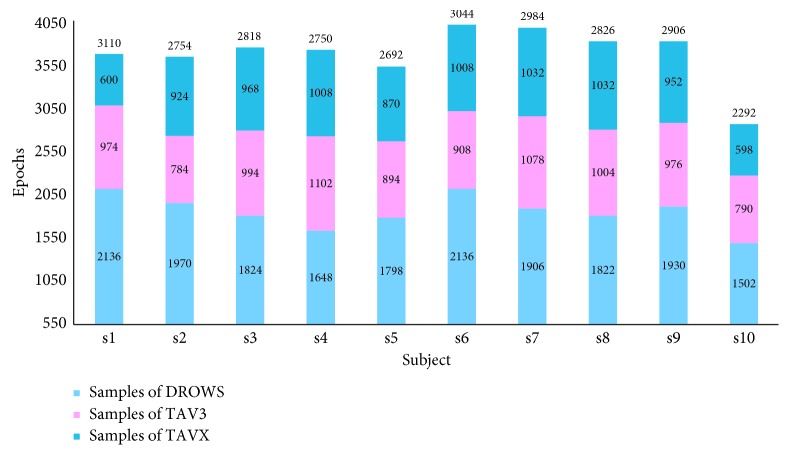
Epoch number of DROWS, TAV3, and TAVX for the subjects.

EEG was recorded by a gUSBamp amplifier with a sampling frequency of 256 Hz and impedance of below 5 k*Ω*. Of 16 channel electrodes, 15 were used to sample EEG, except for ECG sampling heart rate. All the electrodes were referenced to the left earlobe. After removing the artifacts, EEG signals of 15 channels were divided as Fz, Pz, Oz, Fp1, Fp2, F7, F3, F4, F8, C3, C4, P7, P3, P4, and P8, with a time window of 0.5 s, and then a certain number of epochs of each stage were obtained. According to Kar et al. [2], the EEG recording was filtered between 1 and 40 Hz with a band-pass filter, and independent component analysis (ICA) [46] was then adopted for eye movement artifacts rejection. With ICA, the source signal can be separated or approximately separated without knowing the source signalS, noise, and mixing mechanism. After that, according to the method we proposed in [41], EEG recording was converted into SP *∗* CH *∗* TR format, where SP is the sampling frequency, CH is the corresponding channel, and TR is the event. For the segmentation of EEG data, we adopted 0.5 s-interval time window to split EEG data of 15 channels into different number of epochs. Due to the sampling frequency of 256 Hz, we then expressed every epoch as a 15 *∗* 128 matrix. At the same time, we used the flag “0” for DROWS, “1” for TAV3, and “2” for TAVX, respectively. Thus, we obtained a total of 37,168 epochs, including 18,672 DOWNS epochs, 9,504 TAV3 epochs, and 8,992 TAVX epochs, as shown in Figure 3. In this way, LightFD can be trained by these epochs, and the classification performance of LightFD for mental states prediction could be tested simultaneously as well.

## 3. Method

### 3.1. EEG Feature Extraction by Improved CSP

The core of CSP is to find the optimal spatial projection to maximize the power of the two types of signals, so it can estimate two spatial filters to extract the task-related signal components and remove the task-independent components and noise. The method used by CSP is based on the simultaneous diagonalization of two covariance matrices.

For EEG data we extracted, each trail can be represented as a matrix *W* of *X* × *S*, where *X* is the number of channels and *S* is the number of sampling points for each channel. The regularized spatial covariance is shown in the following equation:

(1) $$\begin{array}{c} C=\frac{WW^{T}}{\text{trace}\left(WW^{T}\right)}, \end{array}$$

where trace(·) represents the sum of the diagonal elements of the matrix. In order to separate the two types of variances, we averaged the sum of the covariances of the two types of samples in the training data to achieve the respective average covariances *C*_*d*_ and *C*_*t*_ and then obtained the mixed spatial covariance as *C*_*c*_ = *C*_*d*_+*C*_*t*_. *C*_*c*_, which was decomposed into the form *C*_*c*_ = *E*_*c*_*λ*_*c*_*E*_*c*_, where *E*_*c*_ is the eigenvector of the matrix and *λ*_*c*_ is the diagonal matrix formed by the eigenvalues. The eigenvalues were arranged in descending order, and the whitening transformation was performed according to the following equation:

(2) $$\begin{array}{c} P=\sqrt{\lambda_{c}^{-1}}E_{c}^{T}. \end{array}$$

The eigenvalue corresponding to *PC*_*c*_*P*^*T*^ is 1, so *C*_*d*_ and *C*_*t*_ were transformed as follows: *S*_*d*_=*PC*_*d*_*P*^*T*^, *S*_*t*_=*PC*_*t*_*P*^*T*^. Then, *S*_*d*_ and *S*_*t*_ share common feature vectors; when *S*_*d*_=*Bλ*_*d*_*B*^*T*^, there are *S*_*t*_=*Bλ*_*t*_*B*^*T*^ and *λd*+*λt*=*I*, where *I* is the unit vector matrix. Because the sum of the corresponding two eigenvalues is always 1, when eigenvector *B* has the largest eigenvalue for *S*_*d*_, it has the smallest eigenvalue for *S*_*t*_. Thus, the projection matrix obtained was

(3) $$\begin{array}{c} P_{N}={\left(B^{T}P\right)}^{T}. \end{array}$$

Because three states were used in the experiment, we designed a feature extraction method for the three categories by CSP. For the awake state that was easier to distinguish; we projected the fatigue state data and the neutral state data separately and obtained the projection matrices *P*_*A*_ and *P*_*B*_. Our final projection matrix was

(4) $$\begin{array}{c} P_{N}=P_{A}+P_{B}. \end{array}$$

All experimental samples (including training and testing) were decomposed according to equation (4) to obtain the required EEG characteristics:

(5) $$\begin{array}{c} F=P_{N}W. \end{array}$$

**Figure 4:**
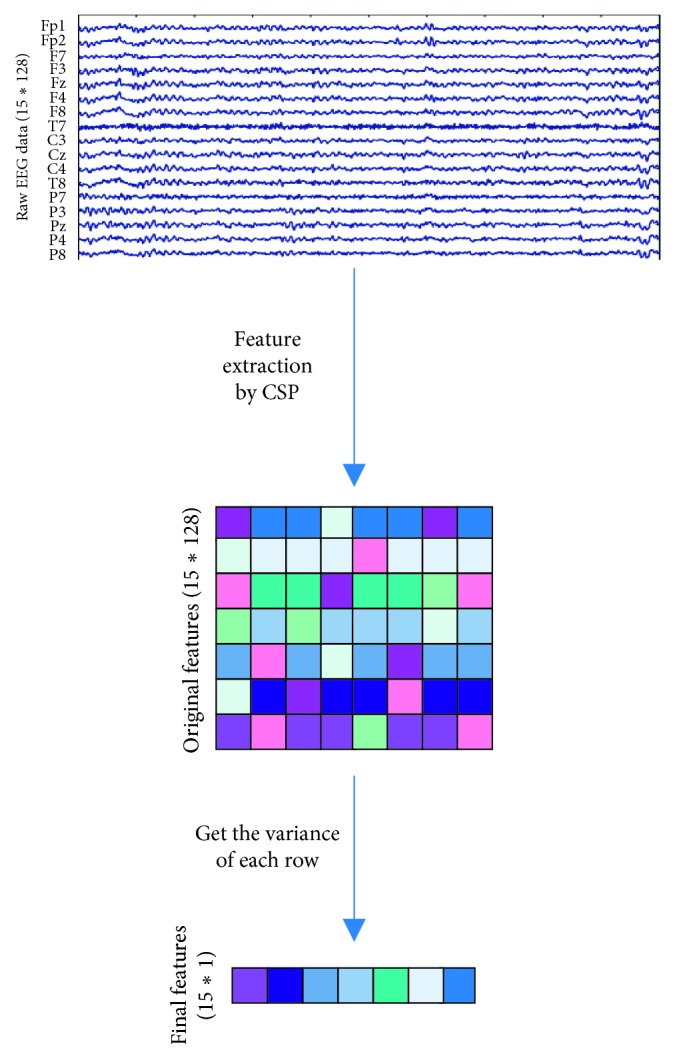
EEG data feature extraction process.

The process of EEG feature extraction is shown in Figure 4. In addition, the high dimensionality of EEG data increased the time and space consumption in deep learning models. But through our experimental tests, we found that LightGBM did not rely on high-dimensional data features as deep learning models. After feature reduction, the training speed was faster, memory consumption was reduced, and the final accuracy did not change much.

Traditional CSP usually uses log variance for feature normalization in the binary-classification problem. While in our proposed improved CSP for EEG-based triclassification problem, after obtaining the feature matrix through the projection matrix *W*, instead of using the conventional method, we used the channel variance of the feature matrix to achieve the purpose of dimensionality reduction. At last, the variance function: var, for each sample, was used to calculate the variance of the data in each channel and reduced the dimensionality of EEG data. Based on this improved CSP, we designed and implemented a LightGBM-based model, LightFD, for the triclassification of driver mental states.

### 3.2. Training of LightFD Classifier

**Figure 5:**
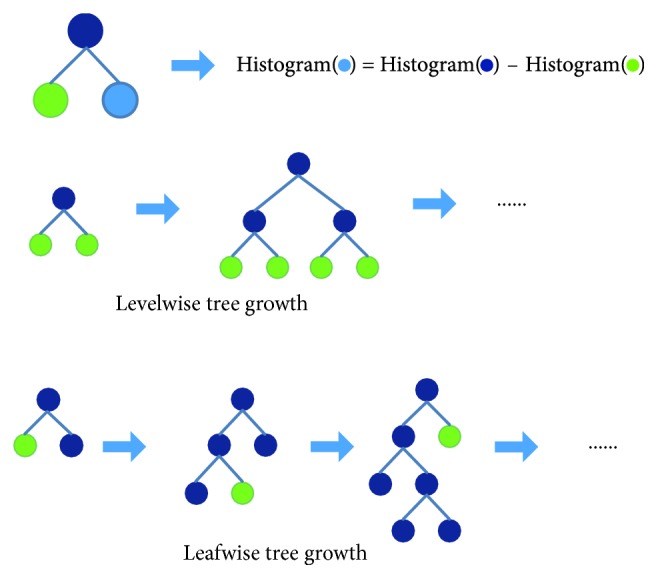
Learning process of LightGBM.

LightGBM is an algorithm for classification that relies on the gradient hoist, and it is known for its light computational burden [33]. In particular, in the tree-based boosting family of algorithms, many of them (such as xgboost) use the presorting algorithm to select and split features. However, this presorting algorithm can accurately find the splitting point, but it has a large overhead in time and memory consumption. The proposed LightFD model adopts histogram algorithm and leaf growth strategy of leafwise with depth limitation, as shown in Figure 5, which can increase computing efficiency, decrease memory occupancy, improve classification accuracy, and prevent overfitting efficiently (please refer to [33, 47] for more detail). The detailed procedure of LightFD is listed as follows.

#### 3.2.1. Histogram Algorithm

The basic idea of the histogram algorithm is to discretize successive floating-point eigenvalues into *k* integers and construct a histogram of width *k*. When traversing the data, the statistic is accumulated in the histogram according to the discretized value as an index. After traversing the data once, the histogram accumulates the required statistic and then traverses to find the optimal segmentation point according to the discrete value of the histogram.

#### 3.2.2. Leafwise Leaf Growth Strategy with Depth Limitation

Levelwise data can split the leaves of the same layer at the same time, easy to multithread optimization, control model complexity. But levelwise is actually an inefficient algorithm because it treats the leaves of the same layer indiscriminately, which brings a lot of unnecessary overhead, and is difficult to prevent overfitting, due to the lower split gain of many leaves, which does not need to be searched and split.

Leafwise strategy is more efficient. It is just to find the leaf that has the highest split gain from the current layer to split. Therefore, compared with levelwise method, leafwise strategy can obtain better performance at the situation with the same number of split. But leafwise strategy may cause deeper decision tree and then be overfitting. So to avoid the situation of overfitting and ensure higher efficiency, we then make a maximum depth limitation in LightFD model.

### 3.3. Parameters of LightFD

The parameters in lightFD include num_leaves, num_trees, and learning_rate, where num_trees represents the total number of spanning trees and num_leaves represents the number of leaves on per spanning tree. Smaller learning_rate and larger num_trees can improve the final accuracy to a certain extent, but it increases the time and space overhead.

## 4. Results and Discussion

As traditional machine learning methods, SVM [48] and LMNN [49] are classical methods for the classification of samples. Deep learning (DL) [50] has been successfully applied in many fields such as computer vision, speech recognition, and natural language processing. LSTM is proposed to overcome the fact that the recurrent neural network (RNN) does not handle long-range dependencies well, although GRU is a variant of LSTM. GRU maintains the effects of LSTM with a simpler structure and plays its own advantages in more and more fields. CNN is a neural network designed to process data similar to grid structures, such as time series data and image data, which has become one of the most important representatives of DL because of its excellent classification performance in many challenging applications [51–53].

In this section, we compare LightFD with SVM, LMNN, GRU, and CNN from both aspects of intrasubject and intersubject. Particularly, because GRU and CNN rely on high-dimensional features, we do not perform dimensionality reduction after CSP but directly use those high-dimensional features as input to GRU and CNN models for training and testing.

For SVM, the kernel type is used with a Gaussian kernel function, the penalty parameter is set to 1.5, and probability estimation is set as “not enabled.” For LMNN, we chose the Euclidean distance as the distance metric, and the nearest neighbor is set to 3. For GRU, we used a single-layer structure with a time step of 128, a learning rate of 0.001, and the RMSprop model as the gradient descent method. For CNN, the model structure contains a 5 × 5 convolutional layer (output number is 32) and a 3 × 3 convolutional layer (output number is 32 as well), followed by a maximum pooling layer with a step size of 2, and a learning rate of 0.01.

### 4.1. Intrasubject Classification Performance

For each subject, we randomly extracted 80% of EEG signals as a training set, denoted as Train_*i*, and the remaining 20% as a test set, denoted as Test_*i*, where *i* = 1, 2,…, 10, indicating the *i*-th subject, the ratio of the training set to the test set is strictly 4 : 1; both Train_*i* and Test_*i* were the data sets after dimensional reduction by our improved CSP.

When comparing LightFD with SVM and LMNN, we adopted Train_*i* and Test_*i* as the training set and test set, respectively, for the analysis of classification performance. Although comparing LightFD with GRU and CNN, due to the high-dimensional feature correlation of GRU and CNN, we did not adopt those features processed by the improved CSP as input but the original data after preprocessing.

**Figure 6:**
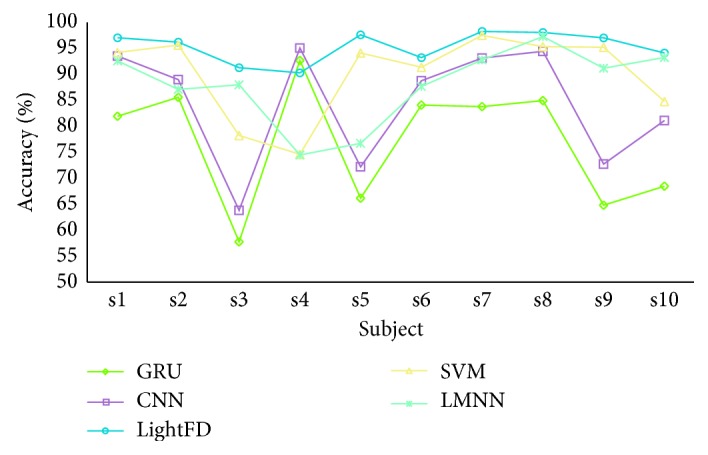
Accuracy comparison of SVM, LMNN, CNN, GRU, and lightFD for intrasubject classification.

The test results are shown in Figure 6. For the mental state detection of the same subject (intrasubject), SVM and LMNN models have similar classification performance, and their average classification accuracy are 90.10% and 88.10%, respectively; however, LightFD reaches the average accuracy of 95.31%, which is much higher than others. GRU and CNN only surpass LightFD in the classification performance of subject s4, whereas the classification accuracy of the other 9 subjects is inferior to that of LightFD.

In addition, we also counted the average classification accuracy of those 5 models for intrasubject, as shown in Table 2. We found that LightFD has the best classification performance among these models.

To evaluate the stability performance of LightFD, we then calculated the variance of the accuracy of LightFD, SVM, LMNN, GRU, and CNN, respectively, as shown in Table 3.

From Table 3, it is clear that SVM and LMNN have some extent of similar stability and better than GRU and CNN, but the variance of LightFD is significantly lower than those of all others, which shows that LightFD has better robustness in EEG signal processing and further lays the foundation for its real application.

**Figure 7:**
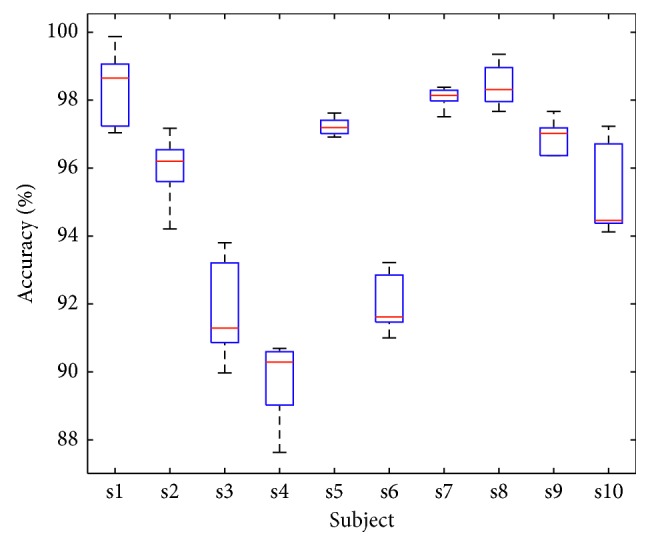
Classification accuracy statistics of 10 subjects under the condition of different testing sets.

Moreover, to validate the applicability of LightFD, we randomly divided the existing data sets for 5 times and obtained 5 groups of data containing different test sets and training sets, then tested the performance of LightFD by the 5 groups of training and test sets. The acquired results are shown in Figure 7. From Figure 7, it is clear that the different data set has a certain impact on the classification result, for example, the average accuracy of subject s4 decreases to about 88%, but in a whole, LightFD keeps a much higher classification accuracy under different test data sets.

### 4.2. Intersubject Classification Performance

EEG signals vary widely among subjects, and these differences can affect the final classification results. To further test the performance of LightFD, in this section, we made a classification performance analysis of intersubject.

Similarly, we mixed all the EEG data of 10 subjects and randomly selected 80% of them as training sets, the remaining 20% as test sets. We also conducted the classification performance analysis and comparison for intersubject analysis between SVM, LMNN, GRU, CNN, and LightFD. To satisfy the input need of GRU and CNN, we do not yet carry out the operation of dimensionality reduction for the two models.

**Figure 8:**
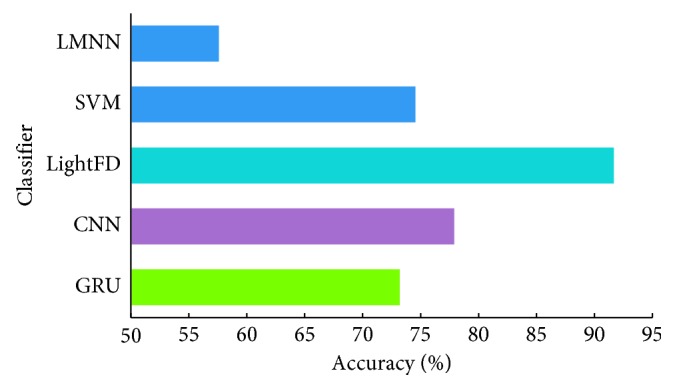
Classification accuracy of SVM, LMNN, and lightFD for intersubject.

As shown in Figure 8, LightFD has a classification accuracy of 91.67%, which is significantly higher than SVM with 74.54%, LMNN with 57.59%, GRU with 73.19%, and CNN with 77.89%. The comprehensive performance of CNN for intersubject analysis is slightly better than SVM but much lower than LightFD. Also from the intersubject classification results, it was found that, compared with intrasubject test, LightFD could maintain more stable performance for intrasubject analysis, although the individual differences of EEG have a greater impact on the classification of the other four models. Therefore, we conclude that LightFD can learn more features and can be better extended to the mental state detection of intersubject.

In addition, similar operation with intrasubject analysis, we could get 5 groups of data with different training sets and test sets, then we also calculated and acquired the average accuracy of each of the three states using these 5 groups of data sets, which are TAV3 95.58%, DROWS 93.97%, and TAVX 83.71%. We found that the classification accuracy of TAVX is low. The reason may be that, according to statistics, the TAVX state is more likely to be misclassified into the state of DROWS.

### 4.3. Transfer Learning Capabilities Analysis of LightFD

Mathematically, transfer learning is defined as below [54].

Given a source domain *D*_*s*_={*X*_*S*_, *f*_*S*_(*x*)} and learning task *T*_*S*_, a target domain *D*_*T*_={*X*_*T*_, *f*_*T*_(*X*)} and learning task *T*_*T*_, transfer learning aims to help improve the learning of the target predictive function *f*_*T*_(·) in *D*_*T*_ using the knowledge in *D*_*S*_ and *T*_*S*_, where *D*_*S*_ ≠ *D*_*T*_, or *T*_*S*_ ≠ *T*_*T*_.

Transfer learning emphasizes on the ability of a system to recognize and apply knowledge and skills learned in previous source tasks transferring to a target prediction tasks.

In this section, we try to evaluate the capabilities of transfer learning of LightFD. In particular, we wanted to measure the performance of LightFD as a general model for real time and efficient driver fatigue detection, which could be directly used for mental states identification without any additional training process. Such last feature could be very important for promoting clinical application of such EEG analysis.

As we know, there exists significant differences of EEG signals between different subjects. Therefore, it is difficult to evaluate the situation of mental states of the other subjects just from EEG characteristics of some known subjects, which means it needs to enhance the transfer learning capabilities in EEG analysis.

First, we selected EEG data from subject s1 to s9 as the training set and that of subject s10 as the test set, then we used CSP to find the projection matrix, and the rest of the operations were consistent with those mentioned in 3.1. Based on the above experimental results, we compared and analyzed the transfer learning performance of LightFD, SVM, and LMNN models, respectively.

For the identification of three mental states, namely, TAV3, TAVX, and DROWS, SVM and LMNN have the classification accuracy of 54.95% and 53.04%, respectively, whereas LightFD could reach the promising classification accuracy of 70.28%, which proves the potential of LightFD in the field of EEG analysis for transfer learning.

**Figure 9:**
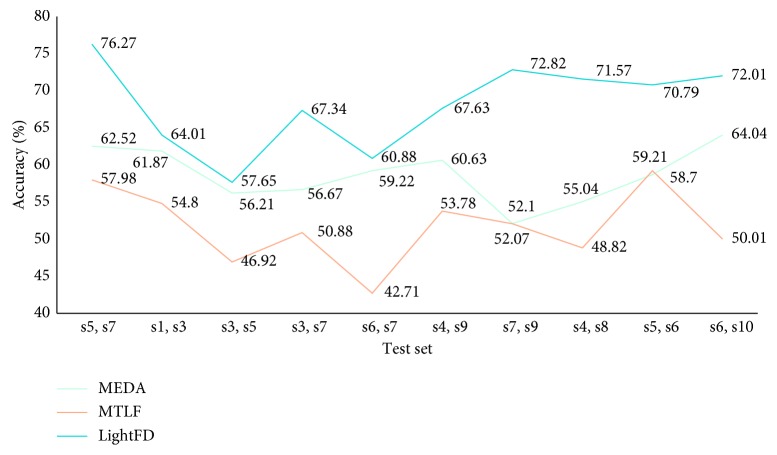
Classification accuracy of MEDA, MTLF, and LightFD for intersubject.

Furthermore, to verify the transfer learning robustness of LightFD, we conducted 10 cross-validations. Of all the ten subjects, we randomly selected two as the test set each time, and the rest as the training set. MEDA and MTLF were used for comparison with LightFD. The randomly selected testing sets for 10 cross-validations are (s5, s7), (s1, s3), (s3, s5), (s3, s7), (s6, s7), (s4, s9), (s7, s9), (s4, s8), (s5, s6), and (s6, s10), respectively, and the results are shown in Figure 9.

In the future, combining with transfer learning will be a major development trend in EEG signal processing. We believe that LightFD, a LightGBM-based model with good performance of EEG transfer learning capabilities, will bring new opportunities and progress for EEG classification and identification analysis.

### 4.4. Time Complexity Analysis of LightFD

In this section, to explore the feasibility of lightFD in practical applications, we analyzed and compared the time complexity of LightFD with the abovementioned 4 typical models: SVM, LMNN, CNN, and GRU.

The obvious benefit of using histogram in lightFD is that the time consumption of calculating the split gain drops from *O*(*N*) to *O*(*bins*). LightGBM usually adopts feature parallelism by vertical segmentation of samples, whereas lightFD adopts sample parallelism, namely, horizontal segmentation, to build local histogram that is then merged into full-range histogram to find the best segmentation. The communication transmission cost is further optimized from *O*(2*∗*#feature*∗*#bin) to *O*(0.5*∗*#feature*∗*#bin).

The time complexity of SVM is between *O*(Nsv^3^+*L*Nsv^2^+*dL*Nsv) and *O*(*dL*^2^), where Nsv is the number of support vectors, *L* is the number of training set samples, and *d* is the dimension of each sample (the original dimension without mapping to the high-dimensional space). In short, its time consumption depends on the matrix inversion, and the time complexity is about *O*(*N*^3^), where *N* is the number of samples. In the case of small samples, SVM can achieve the similar performance as lightFD. But as the number of samples increases, the time consumption of SVM is much higher than that of lightFD.

As a kind of distance metric learning, LMNN needs to calculate the distance between each sample and all other samples during the training process. As the number of samples increases and that of individual sample dimensions grows, it will greatly augment the time consumption of LMNN.

Deep learning models, CNN and GRU, are a kind of high-level abstraction of data by multiple processing layers composed of multiple nonlinear transformations. The complex structure determines that the time complexity is much higher than that of SVM and LMNN, although CNN and GRU tend to perform better when the sample size gets larger and the sample feature dimension becomes higher.

In CNN, the time complexity of single convolutional layer is *O*(*M*^2^ *∗* *K*^2^ *∗* Cin *∗* Cout), where *M* is the size of the output feature map, which is determined by four parameters such as input size *X*, convolution kernel size *K*, padding, and stride. Expressed as follows: *M*=((*X* − *K*+2 *∗* Padding)/Stride)+1. *K* is the size of the convolution kernel, Cin is the number of input channels, and Cout is the number of output channels. It is shown that CNN runs slower and depends heavily on the configuration of the computer under the situation of larger samples.

For GRU, the computational complexity for each update is *O*(*KH*+*KCS*+*HI*+*CSI*)=*O*(*W*), where *K* is the number of output units, *C* is the number of memory element blocks, *S* represents the size of the memory element block, *H* is the number of hidden units, *I* is the number of units that are forward connected to the memory element, the gate unit, and the hidden unit, and *W*=*KH*+*KCS*+*CSI*+2*CI*+*HI*=*O*(*KKH*+*KCS*+*CSI*+*HI*) is the number of weights. GRU is much simpler than CNN and performs better than CNN in case of time consumption. Furthermore, it is faster than SVM and LMNN under the situation of large samples but is still inferior in time consumption than lightFD.

In summary, LightFD has a faster running speed than other traditional models on average up to 30%, which shows more outstanding performance, especially in the case of large samples, and lays the foundation for its application in real-time EEG analysis systems.

## 5. Conclusion

As one kind of light-weighted machine learning methods, LightFD has excellent performance in the aspects of multiclassification of EEG analysis, as well as lower time consumption, which show profound significance for practical applications. In addition, LightFD could also achieve better classification effect in the intersubject EEG classification, which suggests its potential transfer learning capability in the classification of mental states during driving by using cerebral measurements.

## Figures and Tables

**Table 1:** Eight experimental stages.

Stages	Durations (min)	Description
WUP	8–10	Also called “warm-up,” collecting baseline of EEG, ECG, and EOG when driving a car at a predetermined speed.
PERFO	7–9	Also called “performance,” similar to WUP, just has a higher driving speed than that of WUP.

TAV3	7–9	These 5 stages are concurrently exerted task of attention (sound) and video stimuli with different frequency levels. The stages from low to high stimulus frequency are TAV1, TAV2, TAV3, TAV4, TAV5.
TAV1	7–9	
TAV5	6–9	
TAV2	7–9	
TAV4	7–8	

DROWS	12–18	At the stage, the subject falls into drowsiness and feels tired and fatigue.

**Table 2:** Average classification accuracy of SVM, LMNN, GRU, CNN, and LightFD for intrasubject.

Model	SVM	LMNN	LightFD	GRU	CNN
Average accuracy (%)	90.10	88.10	95.31	77.01	84.37

**Table 3:** Variance analysis of SVM, LMNN, GRU, CNN, and LightFD for intrasubject.

Model	SVM	LMNN	LightFD	GRU	CNN
Variance	0.0065	0.0053	0.00084	0.0135	0.0126

## Data Availability

The EEG data used to support the findings of this study are restricted by the Ethics Committee of Hangzhou Dianzi University (HDU), in order to protect subject privacy. Data are available from the corresponding author for researchers who meet the criteria for access to confidential data.
